# Practical Observations on Diseases of the Heart, Lungs, Stomach, Liver, &c., Occasioned by Spinal Irritation; and on the Nervous System in General as a Source of Organic Disease

**Published:** 1836-04

**Authors:** 


### Art. V.

*Practical Observations on Diseases of the Heart, Lungs, Stomach, Liver, &c., occasioned by Spinal Irritation; and on the Nervous System in general as a Source of Organic Disease.* By John Marshall, m.d.—8vo. pp. 172. *London*, 1835.

The subject discussed in this work has often been brought before the public, but without attracting a large or permanent share of its attention. Very many years ago, Dr. Sanders, of Edinburgh, taught his pupils (and perhaps still continues to teach them,) to seek the sources of diseases at the origin of the spinal nerves, and, if we understood right, applied this doctrine in practice; but, so far as we know, never presented it to the world in a formal treatise. Since the commencement, at least, of Dr. Sanders' labours, the Messrs. Griffin, of Limerick, Mr. Tate, Mr. Teale, and others, have written works on affections of the sensitive and motive systems, in which a similar doctrine is advocated, if not with demonstrative proof, certainly with great plausibility; whilst various writers in periodical journals have followed on the same side: among these, Dr. Brown, of Glasgow, published some very valuable observations in the Glasgow Journal; and Mr. Torbet, of Paisley, still more recently, has given some elaborate cases in evidence of the same principle, in the Edinburgh Medical and Surgical Journal for October of last year.

That a doctrine so numerously and ably advocated should have exerted but little influence over the opinions and practice of the medical public, may excite surprise; but a little reflection on the nature of the theory, and the evidence on which it reposes, will, we think, shew why it has failed to impress a very thinking and practical body of men with a conviction of its truth.

In the first place, not only is the nature of what is thought to be the primary pathological condition—the affection designated by the term Spinal Irritation—shadowy and indistinct, but the texture, or part in which it resides, is unknown. Pain is felt in the neighbourhood of the vertebral column. Whence arises this pain? Is it from neuralgia, congestion, inflammation, or something different from all? These questions are unanswered. When we examine its seat, we find it vehemently excited by the slightest touch. This leads inevitably to the conclusion that the situation of the pain thus excited (the spinal marrow may or may not be consentaneously affected,) is external to the vertebral canal: it may be in the ligamentous structure; in the nerves after their exit from the osseous cylinder; or even in the common integument. In a case of the kind we heard of, on undoubted authority, the act of passing the fingers lightly over the fine hairs covering the nape of the neck threw the patient (a lady) into an agony. Here the pain must have been in the skin or the subcutaneous cellular substance.

When we pass to other sources of evidence, we find room for doubt. We admit that the phænomena of the affections ascribed to this irritation are *frequently* of the kind which physiological reasoning would lead us to suppose disease seated at the origin of the spinal nerves calculated to produce. This, however, is not sufficient: we expect some evidence besides those supposed secondary phænomena, of the actual existence of such disease; but this, in most of the cases reported, has been sought in the statements of patients, generally females, labouring under that mixed affection called nervous. In this very sensitive state of the system, we ought certainly to be cautious in drawing inferences regarding pain from the statements of patients, especially if our questions have been put in a leading form. Frequently in such affections, on pressing the spine, the patient will shrink, or even shriek; but when the hand is transferred to another and remote part of the surface, the same indications of uneasiness or suffering will be repeated. No conclusion should be formed as to the existence of spinal irritation, without examining the degree of sensibility in various parts, and comparing it with that over the vertebral column.

Evidence from morbid anatomy, to confirm or refute that deduced from symptoms, is but rarely obtained; cases of this sort being very seldom fatal: but, when death occurs, the author immediately before us declares that no structural change is discernible in the part supposed to be the seat of disease. We admit that symptoms, if clear and unequivocal, are sufficient to establish the existence of a disease; and that, if thus established, it could not be disproved by the absence of perceptible structural change after death; remarking (merely to avoid misconception,) that to suppose a disease, the very essence of which is manifest structural change, —such as phthisis pulmonalis,—to be proved by symptoms to exist, and after death that no such change should be discerned, is to suppose an impossibility. We cannot, however, discover that the evidence, from symptoms, of affection of the spinal marrow, or of the nerves at their origin, is, in the generality of cases, of that conclusive nature to dispense with extrinsic confirmation.

This confirmation has been sought in the result of the treatment adopted: local bleeding, counter-irritation, or both conjointly, directed to the vicinity of the spine. But, in the nervous class (by far the most common) of these affections, it is often so difficult to distinguish between physical amelioration and mental impression, that evidence in favour of the doctrine from this source is, to say the least, of very doubtful validity. Dr. Marshall, who advocates his theory so warmly that, if we understand him right, he would make it almost universally applicable, says, in reference to such measures employed in hysteria, "I have always found it a great matter in such cases to give the patient something to *think about*." Now, it is precisely this "something to think about" which throws such obscurity over our reasonings from the therapeutic measures adopted. When we reflect, moreover, that other remedies, and those such as have been deemed most efficacious in these cases from the days of Sydenham to the present time,—chalybeates and other tonics, for example,—and, above all, change of air and scene, are associated with the local measures, the darkness becomes impenetrable, or the little light perceived shines on the side of the question most remote from the doctrine we are considering. Besides that innumerable cases referred to this class recover without any remedies directed to the supposed seat of the primary affection, the local means, even on the shewing of the warmest advocates of spinal irritation, absolutely require such auxiliaries as change of air and scene for the accomplishment of their object. In one of the cases so instructively reported in the Edinburgh Medical and Surgical Journal, by Mr. Torbet, leeches, blisters, and issues were employed, as some readers will think profusely; but no material improvement took place till the patient was conveyed into Renfrewshire. In another case it is explicitly stated that the complaint was aggravated by an issue in the back; and recovery took place on its healing, the patient being then *in the country*.

It is but justice to the author before us to state, that he does not ask us to accompany him on a voyage solely to this land of shadows: he deals rather in the palpable and distinct: indeed, many of his cases would find a more suitable place in "a Treatise on the Influence of Spinal Distortion on certain Functions of Animal and Organic Life."

The cases, which constitute the great bulk of the volume, are such as the author thinks calculated to illustrate his opinion that various diseases are frequently not idiopathic, but consequent on spinal or ganglionic irritation, or indeed purely indicative of one or the other of these indications. This distinction, however, between disease of an organ caused by spinal irritation, and the mere appearance of such disease indicating irritation of the nervous centre, does not seem to be always kept in view, and the want of its observance is productive of some confusion. The diseases, apparent or real, supposed to be thus induced are, disease of the heart, phthisis, asthma, diseased liver, dyspepsia, cramp in the stomach, diabetes, tabes mesenterica, chorea, and certain non-classified cases.

The very first case does not fall under any of these heads, but, as it shews strongly that, however implicitly we may adopt the facts of an author prepossessed by a theory, the reasoning from them should be our own, we shall give an abstract of it.

"A gentleman, while skating, fell upon the ice, and received a contusion in the lumbar region, from which he did not at first perceive any great inconvenience. He shortly afterwards, however, began to find himself affected with alarming weakness of the lower extremities, and with retention of urine. The weakness rapidly increasing almost to paralysis, the back was examined, and tenderness to touch being found present, the case was treated as a spinal one." (P. 11.) The remedial treatment is not *condescended upon, (sic* in Marshall,) but it is said that considerable amendment in the weakness of the lower extremities took place, but not in the functions of the bladder. In six weeks after the patient came under medical treatment he expired. On examination after death, all the viscera were found healthy, except the kidneys, which were gorged with very dark blood, and contained several small abscesses. No morbid appearance could be detected in the spinal column.

The author's comment on this case is as follows:

" I conceive the *ratio symptomatum* in this case to be, that, at the period the patient fell upon the ice, the renal nerves received an injury or concussion so severe as shortly afterwards to produce paralysis of them. The blood-vessels of the kidneys, and those organs themselves, being thus deprived of nervous energy, became incapable of duly performing their functions. Hence the engorgement of dark blood and breach of structure found after death." (P. 12.)

A more reasonable view of the case would seem to be, that the fall, which produced the injury of the spine, or nerves, indicated by the paralysis, likewise injured the kidneys. The assumption that the change discovered in the latter organs was merely an effect of the lesion of the renal nerves, is purely gratuitous.

The cases of pseudo-disease of the heart are of a miscellaneous character, consisting of affections of various kinds, with only *this* in common, that the medical attendants who preceded Dr. M. had fallen into error in ascribing the symptoms to a disease of the organ itself; whilst, on the other hand, Dr. Marshall has failed, in many of the cases, to adduce adequate proof that the disturbance of the heart's action arose from spinal irritation. Some of the cases, however, in this section shew us the kind and degree of such disturbance, and the disorder in various functions connected with the spinal irritation arising from *deformity*. We select, very much abridged, a few of the cases, both of those which present the strongest evidence of overstrained theory, and those which teach the more valuable lesson, that of endeavouring to discriminate correctly between primary and sympathetic disorder.

"W. E., æt. 12. This case had been pronounced one of disease of the heart, by the ordinary medical attendant and two physicians. His original complaints were weakness, palpitation of the heart, and loss of appetite. He had been for two summers carried to the sea-side, where he uniformly recovered flesh and vigour; but immediately on his return fell off, and experienced a recurrence of all the painful symptoms. Mrs. E. added that he was now worse than he had ever been, and was in fact in so alarming a state as to cause the greatest apprehension of immediate danger. On enquiring what mode of treatment had been pursued, I found that the medical gentleman had most rigidly pursued the starving system of Valsalva for the cure of aneurism; but, instead of Valsalva's bleedings, the boy's bowels had been kept in a constant state of purgation with calomel, jalap, and castor-oil, daily repeated. I could scarcely have believed that the spectre before me was the once blooming, vigorous boy I recollected. He lay in bed emaciated in the extreme, his eyes glistening and restless, moving constantly, with an uneasy rapidity, as if he was unable to fix them for any length of time on the same object. The pulse was sharp, and remarkably quick, 140; the tongue moist, apex florid, but yellow towards the root. The respiration was hurried. The heat of skin rather more than natural. The voice strikingly weak. The expression of the countenance most deplorably anxious. On raising him upon his breech in bed, with a view to examine the chest and spine, I found that the slightest motion increased the rapidity of action of the heart to a most extraordinary degree; and it seemed to beat in every part of the thorax at the same moment. On turning to the spine, *I was disappointed* to find much less irritation or tenderness to the touch than I had anticipated. Drawing the hand down the column gave little or no pain, but caused a thrilling sensation to pass through and over the thorax; this particularly occurred when the hand was passed rapidly down the cervical and dorsal portion of the spine; and at the same time the velocity of the heart's motion was increased far beyond counting; and the patient panted like one who has *ran* (*sic* in original) beyond his strength.

"I used the liberty of altering the treatment completely. I ordered a light, but nutritious diet, to be cautiously substituted for the starving system. The daily purgation to be given up; the bowels to be regulated by a compound rhubarb pill, given at bed-time, *only when required.* The gastric irritation, from which he had suffered so severely, to be treated by the use of bitters and alkalies. Friction along the whole course of the spine and over the thorax with a strong stimulating liniment. In the course of a very few days I had the satisfaction to see a marked improvement arise from this mode of treatment. The friction very soon produced inflammation on the surface; which was quickly followed by a copious eruption; but exactly in proportion as the external irritation along the spine and intercostal nerves increased, the morbid action of the heart and arteries subsided; and the restlessness of the eye, and anxious expression of the countenance, disappeared. By a well-regulated diet, and by the use of the sulphate of iron and quinine, "the bare bones" became quickly covered with a moist comfortable quantity of flesh, his strength and spirits returned, and every symptom of enlarged heart completely vanished." (P. 40.)

The majority of readers will, we think, be disposed to attribute the improvement in this case to the judicious internal remedial and dietetic measures adopted, rather than to the outward application. Simultaneously with this application, it will be observed, improved diet and tonic medicines were in operation, matters of the greatest moment under the deplorable circumstances to which mismanagement had reduced the patient. Disorder of the heart's action arises from sympathy with various parts of the system; but more frequently with the digestive canal than any other. The author acknowledges that, in the present instance, what he calls gastric irritation existed, and disorder of the first passages furnishes a sufficient explanation of all that occurred: the amelioration at the sea-side; the recurrence of distress on quitting it; the pernicious effect of starving and a course of drastic purgatives; and the benefit derived from tonics, a properly regulated diet, and reasonable attention to the bowels, without any reference to the spinal thrill and the spinal friction. So far as the recovery of the patient was concerned, Dr. M. acted the part of a judicious physician, but his doctrine can derive no support from a complication of measures, intended, one would suppose, to bewilder the student, as to what was the essential, what the merely accidental part of the treatment.

The fifth case, one of lateral curvature of the spine, judiciously and successfully treated, is placed among the cases of simulated disease of the heart, for no reason that we can discover, but because palpitation of the organ took place on exertion, an ordinary occurrence in all diseases of debility. With equal propriety it might have been styled simulated dyspepsia or chlorosis, and with much greater, curvature of the spine. Indeed we are much struck by the extreme slightness of the connexion between the symptoms of his simulated diseases and their designation and position in his book. The sixth case we transcribe, not because we think it rare, but as furnishing a good example of the disturbance of various organs connected with spinal deformity.

"James Baillie, aged fourteen. Is reported to have been a very stout child till his fifth year, when he had hooping-cough. From this period he never recovered his health or strength. Was afflicted with breathlessness, palpitation of the heart, and general debility, particularly in the lower extremities; is very small of his age, and has a vacant expression of countenance.

"I found the chest gibbous and ill-developed, the spine forming a posterior curve from the cervical to the bottom of the dorsal vertebræ; the whole length of the column slightly painful to touch. The poor boy is extremely asthmatic, and the palpitation of the heart on taking exercise becomes so violent as to make the head vibrate in unison with its throbbing: bowels torpid; appetite poor; is neither emaciated nor sallow. I suggested that a blister should be put upon the back, and kept open as an issue for a length of time. This was done, and the issue continued for five months: it was then healed, and again in two months was renewed, as his general health seemed to have derived some slight advantage from the former one, and he had grown considerably in the interval. It was kept open for four or five months, but no attention was paid to the other and more important part of the treatment, that of keeping the patient in a recumbent attitude, and preventing any undue exertion. At the end of a year I could not observe any change *to the better upon him* (sic), except that he walked with less difficulty. The irritability of the heart was in no way abated, neither was the asthma. His death was sudden, and appeared to have taken place in a convulsion." (P. 49.)

Dr. Marshall introduces his cases of supposed phthisis by some prefatory remarks, illustrative of his views regarding the pathology of the disease, the original seat of which, he says, is in the nervous system; and certainly, if we admit his reasoning in all its parts, not only is this true of consumption, but of every other disease. He assumes as an undeniable fact, "that it is from the nervous system alone the vascular derives the vital energy by which it performs its functions; and that hence debility or irritation at the root, or in the course of a nerve or set of nerves, must inevitably produce morbid action in the tissues on which they are ramified; whether that be the heart and blood-vessels, the lymphatics, absorbents, or any of the viscera, the effect will be the same, though the particular character of the disease developed will depend upon the seat of it, as well as upon many collateral circumstances." (P. 55.)

Assertions of this kind, which, aiming to illuminate too much, throw a useful light on nothing, are but little to the taste of the present day, essentially utilitarian in character, disposed in some cases to dive deep into science, but according to a precise method and for a practical purpose. The slight and overstrained evidence by which the author attempts to support this theory as applied to phthisis is not more calculated, than its own inherent usefulness, to win for it any share of public approbation. "Neglected catarrh," he says, "usually denominated 'a slight cold,' sudden alternations of temperature; grief, disappointment, and anxiety of mind, especially when preying on it in secret, imprudent over exertion of either the bodily or mental powers, are among the principal causes to which we generally hear consumptive patients or their friends ascribe the commencement of their complaints. I do not at this moment recollect a single instance in which the patient did not mention or readily admit that, among the first unpleasant sensations he could recollect, was that of coldness over the whole body, but *more particularly down the back*." (P. 56.) The italics are the author's own; and the application he intends to be made of the passage thus distinguished is too obvious to require to be indicated.

On examining the cases reported, we find that, by treatment directed to the spine, change of air, &c. five recovered, which were designated phthisis, because cough was associated with vertebral affection; but that the only two really meriting the name were fatal.

In the only case of asthma successfully treated, the evidence of spinal affection is slight, consisting, on the application of the hand to the dorsal vertebræ, in a gasp, like that caused by a sudden plunge into cold water, the whole column being apparently healthy, and the same pressure that caused the gasping causing no pain; whilst little or no confirmation is derived, as it appears to us, from the remedial measures adopted. These consisted in the application of two dozen leeches to the second, third, and fourth dorsal vertebræ, to be repeated every few days, and subsequently blisters in the same situation to be kept open as an issue. Those who are aware how generally asthma has its source in bronchial inflammation, and reflect that these powerful antiphlogistic and derivative remedies were directed to the vicinity of the root of the lungs, will have little difficulty in perceiving how feebly the therapeutic measures tend to supply the defective evidence from other sources. The other case is more important, there being actual distortion, and, moreover, the paroxysms occurring generally when the patient "was pulling on his coat in the morning." This latter circumstance is, however, explicable on another principle than that of the exertion described increasing the spinal irritation. The case was fatal "by the bursting of a blood-vessel in the lungs, causing instant suffocation;" a circumstance which certainly tends to show that there was either disease of the heart, or some hyperemia or other morbid condition of the respiratory organs themselves, and to render in no small degree equivocal the spinal origin of the asthmatic attacks.

Passing over the chapters on dyspepsia and liver disease, but referring our readers to them for cases not always conclusive with regard to the purpose for which they are quoted, yet valuable on account of the general therapeutic treatment with which the more favoured measures are associated, and which in Dr. M.'s hands is always very judicious, we pause upon a solitary case of diabetes.

The circumstances of the case, when first brought under treatment are thus detailed by the author.

"He was brought from his own house to mine, a distance of about seven miles, seated on horseback, where he was held by one of his brothers, his debility being so great as to disable him from otherwise retaining his seat. His appearance truly deplorable; the athletic form wasted to a skeleton; the skin dry and harshy, sallow, almost approaching to a brownish hue. The voice feeble, the respiration hurried; the pulse quick and thready; tongue foul and slimy. Bowels torpid; the eyes sunk in their sockets; the appetite voracious; thirst urgent. Generally voids from four to five gallons of urine during twelve hours, at least during the night; has never exactly ascertained the quantity during the day; but thinks it less than at night. The urine limpid and sweetish-tasted. Thinks himself a dying man." (P. 91.)

After trying, to no beneficial purpose, the tincture of muriate of iron, our author, impressed with the idea "that diabetes originated in irritation or debility of the nerves supplying the stomach and renal apparatus," resorted to frictions of the spine with ointment of emetic tartar. The effect is thus described.

"A copious crop of pustules was produced along the whole length of the back, from the cervix to the sacrum, and shortly after their appearance the symptoms began to subside. The quantity of urine gradually diminished, and became saltish instead of sweet; the appetite became natural in the same proportion; the thirst abated, and the flesh and strength augmented so rapidly, that in a few weeks he walked from his own house a distance of seven miles to visit me, and returned the same day with little fatigue. By the middle of May he was completely out of my hands, and had returned to his ordinary avocations as a farmer." (P. 92.)

This is a result infinitely more favorable than we should have expected from this, or any plan of treatment, unassociated with those dietetic arrangements which are notoriously so important in diabetes. Regarding the adoption or neglect of such arrangements, Dr. M. is totally silent. It is but a solitary case, and the only reasonable inference from it is, that the practice is entitled to a further trial. It may be said, that the ointment acted by establishing a counter-irritation in the vicinity of the kidneys; but our experience of the unsatisfactory result of such derivation in the disease in question forbids our acquiescing in the explanation.

Did our limits permit, it would be easy to show that our author applies his very partial pathology to various diseases, which we have not named, on very slight grounds, and to phlegmasia dolens, which "he is decidedly convinced is of purely nervous origin," for no reason that we can discover. But it is an ungracious task to point out the errors into which an all-engrossing theory has led a man of information and talent. We think it quite supposable that a very refined analysis might discover, at the commencement of almost all diseases, an impression on the sentient part of the frame; but as the majority of the phænomena with which we are practically conversant spring from either a simultaneous or secondary affection of other systems, especially the vascular and secernent, we cannot help regarding an exclusively nervous pathology as of very trifling value. We should indeed regard it as a great misfortune to be obliged in the treatment of disease to direct our attention exclusively to a system with the morbid conditions of which we are so imperfectly acquainted, and over which we have so little control.

Dr. Marshall confidently anticipates "that the time is at hand, when all the rubbish of "anomalous cases," and "intractable and *mysterious* diseases," will be swept from our periodical literature; and when "systems of nosology" only calculated to obscure the subject they pretend to illustrate, will be left to rot unnoticed and undisturbed on the most inaccessible shelves of our libraries." We cannot refrain from the suggestion that it might hasten the advent of this *millennium*, if Dr. Marshall would endeavour to give a more logical form to his own investigations. In the best part of his work, that in which he relates the effects of visible deformity on various functions, we are often left in the dark as to the share which the dislocation of organs or the pressure upon them has in producing the symptoms; and what should be ascribed to the influence of the osseous deformity on the spinal marrow or the nerves as they issue from it. In the other branch of his subject, which he found obscure and has left so, in which spinal irritation is inferred from *supposed* secondary phænomena, and not from manifest disease of the vertebral column, he has erred either in not limiting his measures to the assumed great original of all the symptoms, and thus proving the doctrine; or, by not omitting these local measures, and, if successful, thus aiding to disprove it. This polypharmacy, evidently the result of a latent want of confidence in his own theory, has deprived the cases relating to this branch of the subject of much of their values; which is the more to be regretted, because the public may be said to have returned the Scotch verdict "not proven" on the evidence furnished by Dr. Marshall's predecessors, and to regard the case as still open, and requiring additional and well-sifted testimony for its final settlement.

